# Development and Validation of a High-Performance Liquid Chromatography–Tandem Mass Spectrometry Method for the Simultaneous Determination of Irinotecan and Its Main Metabolites in Human Plasma and Its Application in a Clinical Pharmacokinetic Study

**DOI:** 10.1371/journal.pone.0118194

**Published:** 2015-02-17

**Authors:** Elena Marangon, Bianca Posocco, Elisa Mazzega, Giuseppe Toffoli

**Affiliations:** CRO, National Cancer Institute, Aviano (PN), Italy; UGent / VIB, BELGIUM

## Abstract

Irinotecan is currently used in several cancer regimens mainly in colorectal cancer (CRC). This drug has a narrow therapeutic range and treatment can lead to side effects, mainly neutropenia and diarrhea, frequently requiring discontinuing or lowering the drug dose. A wide inter-individual variability in irinotecan pharmacokinetic parameters and pharmacodynamics has been reported and associated to patients’ genetic background. In particular, a polymorphism in the *UGT1A1* gene (UGT1A1*28) has been linked to an impaired detoxification of SN-38 (irinotecan active metabolite) to SN-38 glucuronide (SN-38G) leading to increased toxicities. Therefore, therapeutic drug monitoring of irinotecan, SN-38 and SN-38G is recommended to personalize therapy. In order to quantify simultaneously irinotecan and its main metabolites in patients’ plasma, we developed and validated a new, sensitive and specific HPLC–MS/MS method applicable to all irinotecan dosages used in clinic. This method required a small plasma volume, addition of camptothecin as internal standard and simple protein precipitation. Chromatographic separation was done on a Gemini C18 column (3 μM, 100 mm x 2.0 mm) using 0.1% acetic acid/bidistilled water and 0.1% acetic acid/acetonitrile as mobile phases. The mass spectrometer worked with electrospray ionization in positive ion mode and selected reaction monitoring. The standard curves were linear (R^2^ ≥0.9962) over the concentration ranges (10–10000 ng/mL for irinotecan, 1–500 ng/mL for SN-38 and SN-38G and 1–5000 ng/mL for APC) and had good back-calculated accuracy and precision. The intra- and inter-day precision and accuracy, determined on three quality control levels for all the analytes, were always <12.3% and between 89.4% and 113.0%, respectively. Moreover, we evaluated this bioanalytical method by re-analysis of incurred samples as an additional measure of assay reproducibility. This method was successfully applied to a pharmacokinetic study in metastatic CRC patients enrolled in a genotype-guided phase Ib study of irinotecan administered in combination with 5-fluorouracil/leucovorin and bevacizumab.

## Introduction

**Fig 1:**
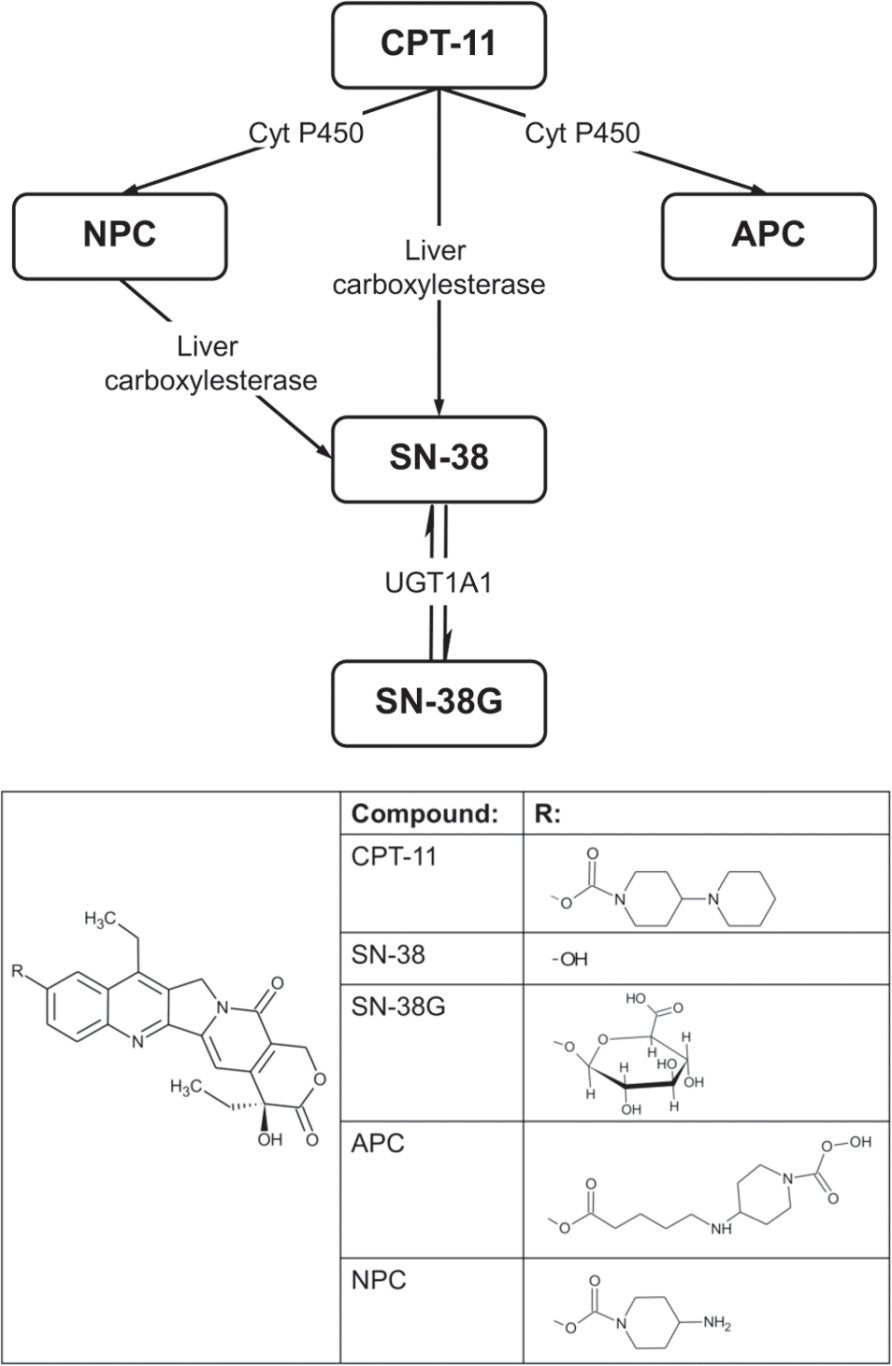
Metabolic pathway and structures of CPT-11 and its main metabolites: SN-38, SN-38G, APC and NPC. Cyt P450: cytochrome P450; UGT1A1: UDP-glucoronosyltransferase 1A1.

Irinotecan (CPT-11) (Fig. 1) is a semisynthetic analogue of the natural alkaloid camptothecin (CPT). It is a prodrug topoisomerase I inhibitor and it is activated by the enzyme carboxylesterase to 7-ethyl-10-hydroxycamptothecin (SN-38) (Fig. 1). Compared with the parent drug, SN-38 is 100- to 1000-times more cytotoxic [1]. Currently, CPT-11 is mainly used in colorectal cancer (CRC) diagnosed patients with metastases, with recorded relapse or progression after application of standard 5-fluorouracil (5-FU)-based therapy [2]. CPT-11 is used as single agent or in combination with other chemotherapeutic (i.e. FOLFIRI: infusional 5-FU, leucovorin, and irinotecan) or biological (i.e. cetuximab and bevacizumab) agents. These regimens include several dosages of CPT-11.

The last 20 years were characterized by the completion of the first sequencing of the human genome and by an intensive development of molecular biology techniques. As a result, the high degree of inter-individual variability was clearly understood and a better knowledge in the biology of cancer was achieved. Recent important advances in the field of pharmacogenetics deserve the inclusion of patient genetic profiling in clinical drug development and in the optimization of antineoplastic chemotherapy. For approved drugs, the dosage recommendations obtained from traditional, non-genotype-directed, phase I studies should be revised in light of validated genetic markers of toxicity risk [3]. The genetic polymorphisms involved in CPT-11 pharmacokinetics (PK) are considered to be reliable predictors of clinical outcome (both for toxicity and efficacy) [4]. In particular, uridine diphosphate glucuronosyltransferase (UGT1A1) is involved in the detossification process of SN-38 by the formation of SN-38 glucuronide (SN-38G). A polymorphism in the *UGT1A1* gene (UGT1A1*28/*28) has been associated to a reduced formation of SN-38G compared with wild-type UGT1A1*1 leading to variability in PK of SN-38 [5–6], and in the toxicity profile of irinotecan [5]. To solve the interindividual variability in PK of irinotecan, Therapeutic Drug Monitoring (TDM) of the parental drug and its metabolites is required. In fact, TDM aims at improving patient care by individually adjusting the dose of drugs and it could improve irinotecan treatment leading to a personalized therapy.

The purpose of this work was to set up and validate a method to perform TDM of irinotecan, quantifying even its main metabolites (SN-38, SN-38G and APC, displayed in Fig. 1) concentrations, in plasma of patients treated with both the standard doses, defined by different treatment protocols, and higher doses as expected in a dose escalation study.

Several methods are currently employed for the determination of irinotecan and its metabolites in human plasma, mainly based on the use of fluorescence detectors, but, to our knowledge, no method is yet available to simply measure irinotecan and, in particular, SN-38G with a high degree of sensitivity and in a wide concentration range. Among these methods, just few could quantify SN-38G but two [7–8] exploit a chromatographic runtime longer than 35 minutes and a too limited range (Owens et al.: 2–25 ng/mL for SN-38, SN-38G and APC and 5–300 ng/mL for CPT-11; Sparreboom et al.: 2–200 ng/mL for all the analytes). Since we mentioned the importance of quantifying SN-38G concentration, the method developed by Sai et al. [9] seemed not suitable because of a SN-38G lower limit of quantification (LLOQ) of 10 ng/mL that appears not fitting to accurately determine the elimination phase of the pharmacokinetic profile of this metabolite.

Among the LC-MS/MS methods, just one, developed by our group, is suitable for simultaneously analyzing CPT-11, SN-38, SN-38G and APC [10] but the upper limit of quantification (ULOQ) (2 μg/mL for CPT-11) seemed not suitable for the quantification of samples collected by patients treated with high-dose irinotecan regimens (> 180 mg/m^2^).

In order to obtain a sensitive, specific and rapid method to quantify irinotecan and its main metabolites in human plasma, we developed and validated, according to the Food Drug and Administration (FDA) and the European Medicines Agency (EMA) guidance on bioanalytical method validation [11–12–13], a high-performance liquid chromatography-tandem mass spectrometry (HPLC-MS/MS) method that requires a small volume of plasma sample (100 μL), only simple treatment with methanol added with acetic acid (0.1% v/v) and a reasonable time of analysis. High selectivity and sensitivity are guaranteed by working in the Selected Reaction Monitoring (SRM) mode [14]. In addition, as we have to quantify irinotecan and its main metabolites on plasma collected by pluri-treated patients, we followed more than one transition for each analyte (one used as quantifier and two as qualifiers), in order to avoid interferences. Lastly, our method was developed using the reference standard of SN-38G avoiding any interferences related to the enzymatic conversion of SN-38.

The present method was successfully applied to a pharmacokinetic study in patients with metastatic CRC enrolled in a genotype-guided phase I study (dose-escalation study) of irinotecan administered in combination with 5-FU/leucovorin (FOLFIRI) and bevacizumab. This guarantees to have the possibility to employ this method to all irinotecan dosages currently used in clinical practice. We investigated a range of concentrations (10–10000 ng/mL for CPT-11, 1–500 ng/mL for SN-38 and SN-38G and 1–5000 ng/mL for APC) that we expected to cover those found in the patients’ plasma even though, on the basis of our signal to noise ratio, we could have validated a lower LLOQ. Moreover, as indicated by the FDA guidelines [12], we evaluated this bioanalytical method by re-analysis of incurred samples as an additional measure of assay reproducibility.

## Experimental

### Standards and chemicals

Analytical reference standards of CPT-11 (7-ethyl-10-[4-(1-piperidino)-1-piperidino]-carbonyloxycamptothecin, batch 059K1163, purity ≥97%), SN-38 (7-ethyl-10-hydroxycamptothecin, batch 088K1267, purity ≥98%) and CPT ((S)-(+)-Camptothecin, batch SLBB9623V, purity ≥90%), used as Internal Standard (IS), were purchased from Sigma-Aldrich Co. (Milan, Italy). APC (7-ethyl-10-[4-N-(5-aminopentanoic acid)-1-piperidino]-carbonyloxycamptothecin, batch 5-PSB-149-1, purity ≥98%) was purchased from Toronto Research Chemicals, Inc. (North York, Ontario, Canada) and SN-38G (7-ethyl-10-[3,4,5-trihydroxy-pyran-2-carboxylic acid]-camptothecin, batch MS0366) was kindly provided by Yakult Honsha Co., Ltd (Tokyo, Japan). Dimethyl sulfoxide (DMSO, batch 037K07663) and LC-MS grade acetonitrile were purchased from Sigma-Aldrich Co. LC-MS grade methanol and acetic acid were supplied by Carlo Erba (Milan, Italy) and Baker (JT Baker, Deventer, NL) respectively. Filtered, deionized water was obtained from a Milli-Q Plus system (Millipore, Billerica, MA, USA). Control human plasma/K_2_EDTA, used to prepare daily standard calibration curves and quality control (QC) samples was provided by the transfusion unit of the National Cancer Institute (Aviano, Italy) from healthy volunteers.

### Standards and quality control solutions

For CPT-11, APC, SN-38 and SN-38G two stock solutions (for standards and quality controls) for each compound were prepared in DMSO at a concentration of 5000.0 μg/mL for CPT-11, 1000.0 μg/mL for APC and 100.0 μg/mL for SN-38 and SN-38G. The stock solution for the IS was prepared at 5 μg/mL in methanol. These solutions were stored at -80°C. A series of working solutions (F to A) to prepare the plasma standard points of the calibration curve and the plasma QC samples (L, M and H) were obtained by mixing and diluting the stock solutions with methanol in order to obtain the final concentrations reported in Table 1. Aliquots of these solutions were kept in polypropylene tubes at -80°C. The IS working solution was prepared at 0.5 μg/mL by diluting the stock solution with methanol.

**Table 1:** Standard and quality control working solutions.

Standards	Concentrations (μg/mL)								
	F	E	D	C	B	A	QCL	QCM	QCH
CPT11	0.20	2.00	20.00	100.00	160.00	200.00	0.50	120.00	180.00
APC	0.02	0.20	2.00	20.00	50.00	100.00	0.04	40.00	80.00
SN38	0.02	0.10	0.50	2.00	5.00	10.00	0.04	3.00	8.00
SN38G	0.02	0.10	0.50	2.00	5.00	10.00	0.04	3.00	8.00

### Preparation of standards and quality control samples

A six-point plasma calibration curve was prepared freshly every day during the validation study. Each calibration sample was prepared by adding 5 μL of the respective standard solution from F to A (ULOQ) to 95 μL of pooled blank human plasma to obtain the final concentrations reported in Table 2. Each calibration curve included a blank sample (plasma processed without IS) and a zero blank sample (plasma processed with the IS). Three QC samples were used for each concentration level. To prepare QC samples, 5.7 mL aliquots of control human plasma were mixed with 300 μL of each working QC solutions (L, M and H) obtaining the QC plasma concentration reported in Table 2. Several 100 μL-aliquots of the three QCs were stored at −80°C to check the analytes stabilities and as controls for future assays. The calibration curve samples and QCs were processed as described below.

**Table 2:** Final concentrations of calibration curve and QC samples.

Standards	Concentrations (ng/mL)								
	F	E	D	C	B	A	QCL	QCM	QCH
CPT11	10.00	100.00	1000.00	5000.00	8000.00	10000.00	25.00	6000.00	9000.00
APC	1.00	10.00	100.00	1000.00	2500.00	5000.00	2.00	2000.00	4000.00
SN38	1.00	5.00	25.00	100.00	250.00	500.00	2.00	150.00	400.00
SN38G	1.00	5.00	25.00	100.00	250.00	500.00	2.00	150.00	400.00

### Processing samples

After thawing plasma samples in an ice bath, they were vortexed for 10 s and centrifuged at 3000 g for 10 min at nominally 4°C. Then 100 μL of the actual sample, standard or QC sample were transferred to a 1.5 mL Eppendorf polypropylene tube, and 5 μL of the IS working solution (0.5 μg/mL) were added and the mixture was vortexed. After that, 300 μL of 0.1%CH_3_COOH/CH_3_OH were added. Each tube was thoroughly vortexed for 10 s and centrifuged at 16000 g for 10 min at nominally 4°C. Then 150 μL of the obtained supernatant were transferred to an autosampler glass vial. Different amounts (3–5 μL), inversely related to the concentrations, were injected into the HPLC system to minimize the carry-over effect. Moreover, after the injection of the ULOQ, three samples of mobile phase and one blank sample were injected to demonstrate the absence of carry-over effect. This procedure guaranteed that no peak higher than 10% of LLOQ was detected. For the same reason, patients’ samples were analyzed on the basis of expected concentrations (lowest to highest), and three samples of mobile phase were injected between successive test samples.

### Chromatographic conditions

The HPLC system consisted of a SIL-20AC XR auto-sampler and LC-20AD UFLC XR pumps (Shimadzu, Tokyo, Japan). Samples were separated on a Gemini C18 chromatographic column (3 μM 110A, 100 x 2.0 mm) coupled with a Security Guard Cartridge (Gemini-NX C18 4.0 x 2.0 mm), both provided by Phenomenex (Torrance, CA, USA) and thermostatically controlled at 25°C. The mobile phases (MP) used for chromatographic separation were 0.1% CH_3_COOH/bidistilled water (MP A) and 0.1% CH_3_COOH/acetonitrile (MP B). The HPLC system was set up with a flow rate of 0.3 mL/min and the following linear gradient:

*step 1*: the initial condition of 95% MP A held for 1 min;

*step 2*: from 95% MP A to 30% over 5.5 min;

*step 3*: constant for 1.5 min;

*step 4*: from 30% MP A to 10% over 0.5 min;

*step 5*: kept constant for 1.5 min;

*step 6*: from 10% MP A to the initial condition over 1 min and reconditioning for 7 min.

The total run time was 18 min.

### Mass spectrometry

The HPLC system was coupled with an API 4000 triple quadrupole mass spectrometer AB SCIEX (Massachusetts, USA). Standard solutions prepared in 0.1% CH_3_COOH acetonitrile/water 1:1 (50 ng/mL) of CPT-11, SN-38, SN-38G, APC and IS were infused at a flow rate of 10 μL/min in order to optimize all the MS parameters. Positive ion mode was used to obtain the mass spectra (MS^1^) and the product ion spectra (MS^2^). The instrument was equipped with a TurboIonSpray source operated at 650°C and with ion spray voltage of 5500 V. The biological samples were analyzed with electrospray ionization (ESI), using zero air as nebulizer gas (30 psi) and as heater gas (65 psi). Nitrogen was employed as curtain gas (20 psi) and as collision gas at medium intensity (CAD). After fragmentation, the characteristic product ions of the five compounds were monitored in the third quadrupole at *m/z* 124.2, *m/z* 167.2 and *m/z* 195.2 for CPT-11, at *m/z* 349.3, *m/z* 249.1 and *m/z* 293.2 for SN-38, at *m/z* 393.2, *m/z* 349.2 and *m/z* 249.2 for SN-38G, at *m/z* 393.3, *m/z* 227.1 and *m/z* 349.2 for APC and at *m/z* 305.1, *m/z* 248.9 and *m/z* 220.1 for IS. Quantification was done in SRM mode using the following transitions: *m*/*z* 587.4 *>* 124.2 for CPT-11, *m*/*z* 393.3 *>* 349.3 for SN-38, *m*/*z* 569.3 *>* 393.2 for SN-38G, *m*/*z* 619.2 *>* 393.3 for APC and *m*/*z* 349.2 *>* 305.1 for the IS (CPT). These transitions were used to define the matrix effect during the method development by means of the post-column infusion. The post-column infusion experiments were performed as follows: standard solutions prepared in 0.1% CH3COOH acetonitrile/water 1:1 (50 ng/mL) for each analyte and IS were infused by a syringe pump during the chromatographic run of an extracted blank human pooled plasma sample. The extracted plasma sample eluted from the HPLC column and the flow from the infusion pump were combined with a zero-dead-volume ‘T’ union and introduced into the source of the mass spectrometer.

To quantify the chromatographic peaks, data were processed with Analyst 1.5.2 (quantification with MultiQuant 2.1) software package (AB SCIEX).

### Validation Study

This study was conducted in accordance with the EMA and the FDA guidance on bio-analytical method validation [11–12–13]. The method was validated by examining the following parameters: recovery, linearity, intra- and inter-day precision and accuracy, reproducibility, limit of detection (LOD), LLOQ, selectivity, matrix effect and stability.

Recovery

The percentage extraction recovery was determined for each analyte (CPT-11, SN-38, SN-38G and APC) at three plasma concentrations (QCL, QCM and QCH) prepared in quintuplicate. The peak area of each analyte extracted from plasma QC samples were compared to those from external standards prepared in methanol. The recovery of IS was evaluated in the same way at a plasma concentration of 25 ng/mL. Moreover, for every analyte and IS, the percentage recovery was even determined by comparing the peak area of the analyte extracted from plasma QC samples, prepared at the three concentrations, with the peak area of the extracted matrix prepared in five replicates and added with the same amount of the analytes (data not shown).

Linearity

The linearity of calibration curves was validated on five different working days with calibration curves prepared as described in the Section on Preparation of Standards and Quality Control samples. For each standard point, the ratio of the HPLC–MS/MS peak area for CPT-11, SN-38, SN-38G and APC to the IS was calculated and plotted against the nominal concentration of each analyte in the sample. The linearity of the standard curves was checked by regression analysis and the goodness of the regression by calculating the Pearson’s determination coefficient R^2^ and by comparison of the true and back-calculated concentrations of the calibration standards. The accuracy of back-calculated values of an individual point had to be within 85–115% of the theoretical concentration (80–120% at the LLOQ), and a minimum of five standards had to meet these criteria, including the LLOQ and highest calibrator, ULOQ [11–12–13].

Intra-day and inter-day precision and accuracy and reproducibility

Precision and accuracy were evaluated on five different days by measuring the analytes in three replicates at three QC levels at the nominal concentrations reported in previous Table 2. To analyze the QC samples, different standard calibration curves were plotted and processed on each of the five days of the validation study. The precision of the method at each concentration was reported as the coefficient of variation (CV%), expressing the standard deviation as a percentage of the mean calculated concentration. The accuracy was determined by expressing the mean calculated concentration as a percentage of the nominal concentration. In each run, the measured concentration for at least six out of nine QC samples had to be within 15% of the nominal value. Only one QC sample could be excluded at each concentration level. As indicated in the FDA Draft Guidance for Industry on Bioanalytical Method Validation -September 2013-(Biopharmaceutics, Revision 1) [12], a revised version of the guidance published in May 2001 [11] taking into account the AAPS/FDA Workshop on Incurred Sample Reanalysis (February 2008) [15], evaluation of bioanalytical methods by re-analysis of incurred samples should be performed and can be considered as an additional measure of assay reproducibility. Incurred Sample Reanalysis (ISR) is a necessary component of bioanalytical method validation and is intended to verify the reliability of the reported subject sample analyte concentrations. ISR is conducted by repeating the analysis, with the same bioanalytical method procedures, of a subset of subject samples from a given study in separate runs on different days to critically support the precision and accuracy measurements established with spiked QCs. Therefore, the accuracy of the present method was assessed by re-analyzing the incurred plasma samples of one patient from the pharmacokinetic study in a further analytical session. The selection of samples for reanalysis was done guaranteeing adequate coverage of the PK profile in its entirety including a sample around the maximum concentration (Cmax) and in the elimination phase. The analyses can be considered equivalent if two-thirds (67%) of the percentage difference [(repeat-original)*100/mean] of the results is within 20%.

Limit of detection, limit of quantification, selectivity and matrix effect

The LOD is the concentration at which the signal-to-noise ratio (S/N) is at least 3. The LLOQ of the bioanalytical method is the concentration of the lowest standard. The analyte response at the LLOQ should be at least 5 times the response compared to blank response. The LLOQ of the present method was assessed by adding F working solution to six samples of blank human plasma, to obtain the final concentration reported in Table 2. Selectivity was proved using six independent sources of blank human plasma, which were individually analyzed and evaluated for interference: a single 95 μL-aliquot from each of the six matrices was spiked with the analytes at the LLOQ [11–12–13]. Both LLOQ and selectivity had to have acceptable accuracy (≤20%) and precision (between 80% and 120%) [11–12–13]. During the validation study the potential for matrix effects on the quantification of CPT-11, SN-38, SN-38G and APC was also tested. Matrix effects arise due to effects of endogenous components of the plasma matrix on the ionization of the analytes of interest and IS. Matrix effects were investigated on six independent sources of blank human plasma, by calculating the ratio of the peak area in the presence of matrix to the peak area in absence of matrix at the three different QC concentrations (L, M and H) of each analyte. The CV should be within 15% [13].

Stability

Plasma stability of CPT-11 and its main metabolites was assessed by analyzing QC samples for each standard at the three different concentrations (L, M and H) during sample storage and handling. Bench-top stability was determined after 2 h at room temperature and in ice bath and the stability of the processed samples in the autosampler by repeatedly analyzing the processed QC samples 24, 48 and 96 h after the first injection. To check freeze/thaw stability, a freshly prepared aliquot of each QC sample concentration was processed and analyzed, and then again after one and two freeze/thaw cycles. Long-term stability was assessed in plasma and in working solutions stored at approximately −80°C. Each analyte was considered stable at each concentration when the differences between the freshly prepared samples and the stability of testing samples did not deviate more than 15% from the nominal concentrations.

Application of the method to clinical samples

The method was used to explore the PK of CPT-11 and its main metabolites in metastatic CRC patients during a phase Ib clinical trial of high-dose irinotecan (260–370 mg/m^2^) administered in FOLFIRI plus bevacizumab regimen. Patients received CPT-11 as a 120 min intravenous infusion on days 1 and 15 of a 28-day treatment cycle. Serial blood samples were collected on days 1–3 and 15–17 of the first cycle of treatment. Samples were collected into tubes containing K_2_-EDTA (as the anticoagulant) at the following time-points: before drug administration, and at 1.0, 2.0, 2.25, 2.50, 3.0, 4.0, 6.0, 8.0, 10.0, 14.0, 26.0, 50.0 h following the start of the irinotecan infusion. Plasma was obtained immediately by centrifugation of the blood samples at 3000 g for 10 min at 4°C. Then the plasma was separated, split into 2 polypropylene tubes and stored as two independent aliquots at -80°C pending analysis. All blood samples were collected under the full ethical approval of the ethics committee of the participating centers and only after the signature of informed consent from all the enrolled patients. The study was approved by the ethics committee of CRO-National Cancer Institute of Aviano (Italy) and of the University of Chicago Medicine (Chicago, IL) and by Istituto Superiore di Sanità (ISS, Italy), Prot. n. 0041793(09)-PRE.21-984. The phase I study was conducted according to the principles expressed in the Declaration of Helsinki.

## Results and Discussion

### HPLC-MS/MS

**Fig 2:**
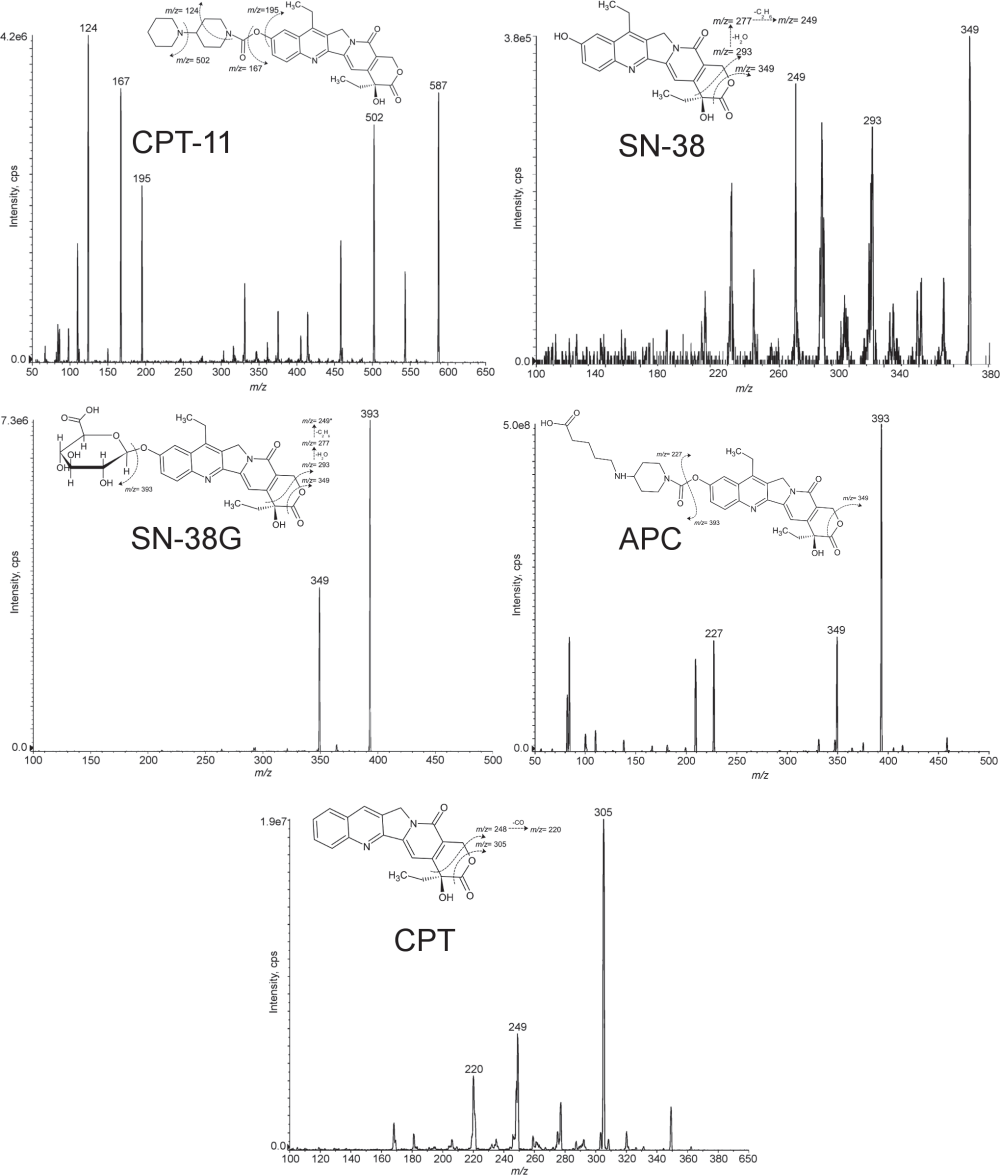
MS/MS mass spectra of CPT-11, SN-38, SN-38G and APC with chemical structures and identification of the main fragment ions. The fragment ion at *m/z* *248 of SN-38G is not shown in the MS/MS mass spectrum because it requires, for its formation, a higher collision energy than the other fragments.

**Fig 3:**
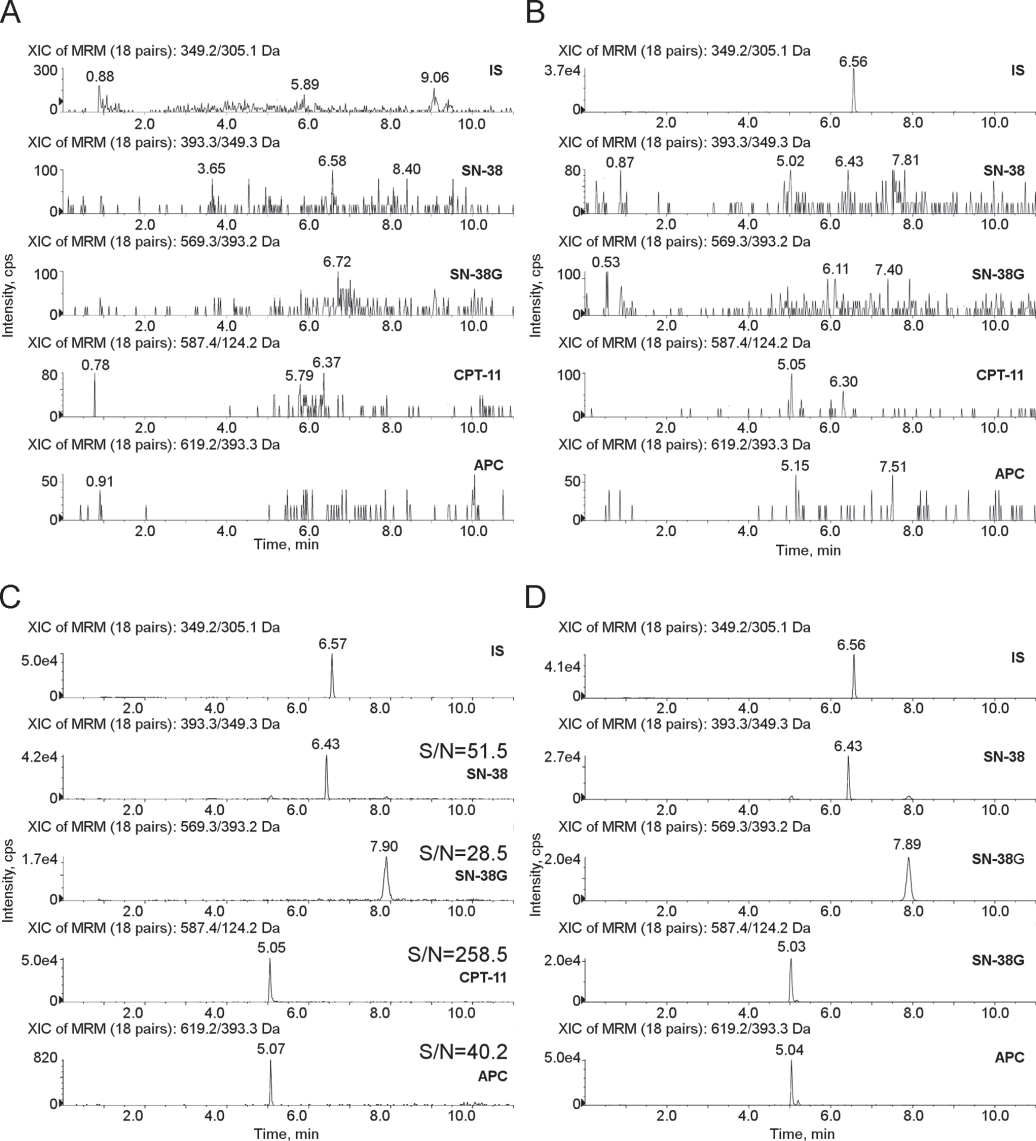
Representative SRM chromatograms. Panel A: SRM chromatograms of a human blank plasma sample; Panel B: SRM chromatograms of a human blank plasma sample with IS added; Panel C: S/N of SN-38, SN-38G, CPT-11 and APC at the LLOQ (10 ng/mL for CPT-11 and 1 ng/mL for SN-38, SN-38G and APC); Panel D: SRM chromatograms of an extracted plasma sample of a treated patient showing IS, SN-38, SN-38G, CPT-11 and APC. The concentrations measured were 7.42, 16.57, 80.27 and 12.91 ng/mL for SN-38, SN-38G, CPT-11 and APC, respectively.

To optimize the mass spectrometer conditions, an infusion of each standard solution at 50 ng/mL in mobile phases (50:50) was used. Using an ESI source in positive ion mode, irinotecan and its main metabolites formed mainly a protonated molecule [M+H]^+^. The precursor ion of CPT-11, SN-38, SN-38G, APC and CPT as IS (*m*/*z* 587.4, *m*/*z* 393.3, *m*/*z* 569.3, *m*/*z* 619.2 and *m*/*z* 349.2 respectively) passed through the first quadrupole into the collision cell and the collision energy (CE) was optimized to obtain their product ions with a high signal. After fragmentation, the characteristic product ions of the five compounds were monitored in the third quadrupole at *m/z* 124.2 (51 V), *m/z* 167.2 (58 V) and *m/z* 195.2 (44 V) for CPT-11, at *m/z* 349.3 (35 V), *m/z* 249.1 (68 V) and *m/z* 293.2 (47 V) for SN-38, at *m/z* 393.2 (40 V), *m/z* 349.2 (60 V) and *m/z* 249.2 (104 V) for SN-38G, at *m/z* 393.3 (45 V), *m/z* 227.1 (36 V) and *m/z* 349.2 (62 V) for APC and at *m/z* 305.1 (33 V), *m/z* 248.9 (43 V) and *m/z* 220.1 (48 V) for IS (Table 3). The fragmentation patterns are represented in Fig. 2. For each compound, the daughter ion with the highest signal was used as quantifier, as follows: 587.4 >124.2 for CPT-11, 393.3>349.3 for SN-38, 569.3>393.2 for SN-38G, 619.2>393.3 for APC and 349.2>305.1 for IS, all expressed in *m/z*. Fig. 3 presents typical SRM chromatograms, using the quantifier transitions noted above. Panel A shows an extracted blank plasma sample; Panel B displays an extracted blank plasma sample with IS added; Panel C shows an extracted plasma sample at the LLOQ with IS added and Panel D displays an extracted plasma sample of a patient, drawn 26 h after the drug dose of 310 mg/m^2^. The peaks correspond to a concentration of 80.27, 7.42, 16.57 and 12.91 ng/mL of CPT-11, SN-38, SN-38G, APC, respectively. The elution of the analytes was rapid and selective with adequate separation of all the peaks within 9 min: CPT-11, SN-38, SN-38G, APC and IS were eluted at approximately 5.05, 6.43, 7.9, 5.07 and 6.57 min, respectively. No interfering peaks were observed at these retention times, and the peaks were completely resolved from plasma matrix, with a good shape. The specificity of the method was confirmed by analysing six independent sources of blank human plasma.

**Table 3:** Source- and compound-dependent parameters and ion transitions of each analyte and IS used for the mass spectrometer method.

Precursor Ion				Daughter Ion		
Analyte	Q1 (amu)	DP (volts)	EP (volts)	Q3 (amu)	CE (volts)	CXP (volts)
CPT-11	587.4	125	11	124.2	51	6
				167.2	58	10
				195.2	44	13
SN-38	393.3	103	13	349.3	35	8
				249.1	68	15
				293.2	47	13
SN-38G	569.3	113	11	393.2	40	8
				349.2	60	7
				249.2	104	16
APC	619.2	115	12	393.3	45	9
				227.1	36	14
				349.2	62	7
CPT (IS)	349.2	75	10	305.1	33	15
				248.9	43	16
				220.1	48	13

The dwell time of each transition was set up at 50 msec. DP: declustering potential; EP: entrance potential; CE: collision energy; CXP: collision cell exit potential.

### Validation of the method

Recovery

The extraction method is based on simple deproteinization with three volumes of 0.1%CH_3_COOH/CH_3_OH relative to plasma sample. The recovery, evaluated in five replicates at three QC concentrations, was in the range 66.4–68.8% (CV ≤8.6%) for irinotecan, 77.2–84.1% (CV ≤9.4%) for SN-38, 54.8–58.6% (CV ≤12.1%) for SN-38G and within 44.0–49.1% (CV ≤10.6%) for APC, as shown in Table 4. The recovery of IS was 30.6% (CV 6.9%).

**Table 4:** Recovery of the analytes and the IS from human plasma.

Analyte	Nominal concentration (ng/mL)	Recovery (%) ± SD	CV %
CPT-11	25.00	66.4 ± 5.7	8.6
	6000.00	68.3 ± 1.6	2.3
	9000.00	68.8 ± 1.6	2.3
SN-38	2.00	77.2 ± 7.3	9.4
	150.00	83.8 ± 2.8	3.4
	400.00	84.1 ± 1.0	1.2
SN-38G	2.00	58.6 ± 7.1	12.1
	150.00	54.8 ± 2.6	4.8
	400.00	56.4 ± 0.8	1.4
APC	2.00	44.0 ± 4.7	10.6
	2000.00	47.6 ± 2.3	4.7
	4000.00	49.1 ± 0.9	1.9
CPT (IS)	25.00	30.6 ± 2.1	6.9

Calibration curves

**Fig 4:**
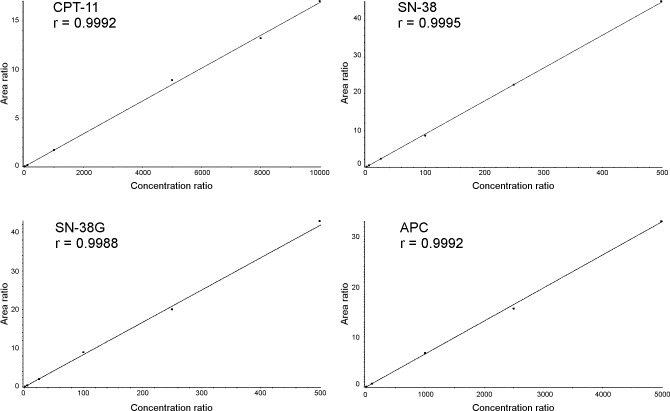
Calibration curve of CPT-11 and its main metabolites SN-38, SN-38G and APC in human plasma.

Table 5 reports the results for the calibration curves of CPT-11 and its main metabolites freshly prepared every day during the validation study, and the accuracy and precision for each standard. The peak-area ratios of the analyte/IS compared to the nominal concentrations were plotted and a least-squares linear regression, weighted by the reciprocal of the concentrations, were plotted and a weighted quadratic regression function (1/x^2^) was applied to generate calibration curves (Fig. 4). The calibration curves prepared on five different days showed good linearity and acceptable results of the back-calculated concentrations over the validated range of 10.00–10000.00 ng/mL for CPT-11, of 1.00–500.00 ng/mL for SN-38 and SN-38G and of 1.00–5000.00 ng/mL for APC. Pearson’s coefficient of determination R^2^ was ≥0.9962 for each run, the mean accuracy was always close to 100% (range 93.4–111.9% for CPT-11, 97.0–102.8% for SN-38, 98.7–101.4% for SN-38G and 94.3–105.4% for APC) and the precision, expressed as CV%, ranged from 0.3% for the lowest calibrator (10.00 ng/mL) to 6.6% for CPT-11, from 1.0 to 6.6% for SN-38, from 1.5 to 7.7% for SN-38G and from 0.7 to 8.4% for APC. As reported in the Section on Processing Samples, the carry-over effect was minimized injecting three samples of mobile phase between successive test samples and after the injection of the ULOQ. This action guaranteed peak response no higher than 10% of LLOQ. In order to quantify patients’ samples, a calibration curve was freshly prepared every analysis run and the samples’ concentrations were back-calculated from the calibration curve.

**Table 5:** Linearity, accuracy and precision data for calibration curves of irinotecan and its main metabolites.

Analyte	Nominal conc. (ng/mL)	Mean ± SD	Precision %	Accuracy %
CPT-11	10.00	9.88 ± 0.03	0.3	98.8
	100.00	111.90 ± 3.18	2.8	111.9
	1000.00	1070.91 ± 61.18	5.7	107.1
	5000.00	4671.81 ± 114.51	2.5	93.4
	8000.00	7568.46 ± 500.11	6.6	94.6
	10000.00	9420.12 ± 513.90	5.5	94.2
SN-38	1.00	1.00 ± 0.01	1.0	99.8
	5.00	5.01 ± 0.29	5.9	100.2
	25.00	25.70 ± 0.86	3.4	102.8
	100.00	99.35 ± 5.09	5.1	99.4
	250.00	251.99 ± 10.01	4.0	100.8
	500.00	484.99 ± 31.86	6.6	97.0
SN-38G	1.00	1.00 ± 0.01	1.5	100.1
	5.00	4.98 ± 0.38	7.7	99.6
	25.00	24.67 ± 0.86	3.5	98.7
	100.00	101.40 ± 3.48	3.4	101.4
	250.00	253.43 ± 14.12	5.6	101.4
	500.00	494.14 ± 34.95	7.1	98.8
APC	1.00	1.00 ± 0.01	0.7	99.7
	10.00	10.38 ± 0.68	6.5	103.8
	100.00	105.44 ± 5.01	4.8	105.4
	1000.00	943.42 ± 39.02	4.1	94.3
	2500.00	2469.92 ± 207.95	8.4	98.8
	5000.00	4935.06 ± 264.42	5.4	98.7

Intra-day and inter-day precision and accuracy and reproducibility

The precision and accuracy of the method were evaluated by analysing three replicates of QC samples (QCL, QCM and QCH) within a single-run analysis for intra-day assessment and over five consecutive runs for inter-day assessment. The accuracy and precision (CV%) obtained are shown in Table 6. The method was very precise, with intra- and inter-day CV ≤8.9% and ≤8.7% for CPT-11, ≤11.6% and ≤9.9% for SN-38, ≤9.0% for SN-38G and ≤10.7% and ≤12.2% for APC. Moreover, the method showed intra- and inter-day accuracy within the range 101.0–106.3% and 96.3–99.8% for CPT-11, 96.2–109.0% and 96.9–105.9% for SN-38, 92.3–113.0% and 98.3–102.3% for SN-38G and 89.4–106.1% and 94.1–103.2% for APC. The good reproducibility and accuracy of the method were further demonstrated by re-analysis of incurred plasma samples of one patient treated at the dose of 260 mg/m^2^. The concentrations of irinotecan and its main metabolites determined on the two analytical runs were very similar in all samples, being the percentage difference of the results within 20% for more than 71% of the total amount of samples re-analyzed. This range encompasses the accepted variability of the analytical method; hence, the two measurements can be considered equivalent.

**Table 6:** Intra and inter-day precision and accuracy of the method for the analysis of irinotecan and its main metabolites in human plasma samples.

	Analyte	Nominal conc. (ng/mL)	Mean ± SD	Precision %	Accuracy %
Intra-day (N = 5)	CPT-11	25.00	25.42 ± 2.26	8.9	101.7
		6000.00	6379.41 ± 520.23	8.2	106.3
		9000.00	9090.55 ± 369.12	4.1	101.0
	SN-38	2.00	2.17 ± 0.14	6.5	108.7
		150.00	163.43 ± 18.97	11.6	109.0
		400.00	384.85 ± 14.03	3.6	96.2
	SN-38G	2.00	1.85 ± 0.17	9.0	92.3
		150.00	169.52 ± 3.73	2.2	113.0
		400.00	405.40 ± 15.15	3.7	101.3
	APC	2.00	2.10 ± 0.22	10.7	105.2
		2000.00	1788.03 ± 83.27	4.7	89.4
		4000.00	4242.35 ± 170.47	4.0	106.1
Inter-day (N = 14)	CPT-11	25.00	24.77 ± 2.16	8.7	99.1
		6000.00	5986.95 ± 483.98	8.1	99.8
		9000.00	8667.00 ± 580.72	6.7	96.3
	SN-38	2.00	2.02 ± 0.20	9.9	101.2
		150.00	158.78 ± 13.97	8.8	105.9
		400.00	387.53 ± 21.46	5.5	96.9
	SN-38G	2.00	1.98 ± 0.18	9.0	98.9
		150.00	153.45 ± 11.89	7.8	102.3
		400.00	393.07 ± 20.94	5.3	98.3
	APC	2.00	2.06 ± 0.25	12.2	103.2
		2000.00	1881.31 ± 158.89	8.4	94.1
		4000.00	3984.00 ± 346.26	8.7	99.6

Limit of detection, limit of quantification, selectivity and matrix effect

The LOD was defined as the concentration at which the S/N was at least 3. The LOD was 58 pg/mL for SN-38, 105 pg/mL for SN-38G, 116 pg/mL for CPT-11 and 75 pg/mL for APC. The LLOQ was defined as the lowest concentration that could be measured with a precision within 20% and accuracy between 80% and 120%. As shown in Panel C of Fig. 3, with the high S/N obtained (S/N range: 28.5–258.5), it would have been possible to fix a lower LLOQ for each analyte. However, the LLOQ values were chosen on the basis of the concentration range expected in plasma samples of patients enrolled in the phase I study. Therefore, the LLOQ was fixed at 1 ng/mL for SN-38, SN-38G and APC and at 10 ng/mL for CPT-11 and was validated through analysis of six replicates. The accuracy and precision at the LLOQ were determined by analyzing six replicates of the sample at the LLOQ concentration. The accuracy and CV% were, respectively, 91.0% and 4.9% for irinotecan, 111.4% and 5.2% for SN-38, 94.2% and 7.9% for SN-38G and 98.3% and 11.2% for APC. The method was not affected by endogenous components in the matrix or other components in the sample; on spiking six different sources of human plasma with irinotecan and its main metabolites at a concentration corresponding to the LLOQ the precision was 4.9, 5.2, 7.9 and 11.2% for CPT-11, SN-38, SN-38G and APC, respectively and the accuracy was 91.0, 111.4, 94.2 and 98.3%, respectively. There were no significant variations (<15%) in the peak area of each analyte in the six lots of matrix, therefore it was possible to exclude the presence of any matrix effect of ion suppression or enhancement.

Stability

The stability of CPT-11 and its main metabolites, under different conditions, was assessed by analyzing QC samples, prepared in triplicate. All these analytes in human plasma were stable for 2 h in ice bath and for 96 h in the autosampler at 4°C after extraction (S1 Table). Irinotecan and its main metabolites were stable in human plasma over two freeze/thaw cycles: precision as CV% and accuracy for freeze/thaw samples were ≤5.3% and within 86.7–97.6% for CPT-11, ≤6.5% and within 101.3–105.4% for SN-38, ≤3.2% and within 88.1–90.7% for SN-38G and ≤11.0% and within 94.6–95.3% for APC (S2 Table). After 4 months of storage in human plasma, at approximately -80°C, precision (CV%) and accuracy obtained were ≤9.9% and within 94.4–102.2% for CPT-11, ≤8.3% and within 93.5–103.6% for SN-38, ≤8.6% and within 85.2–92.0% for SN-38G and ≤8.3% and within 92.5–104.5% for APC (S2 Table). The standard working solutions of CPT-11, SN-38, SN-38G and APC used for calibration curve and QC samples, prepared in methanol and stored at −80°C, were stable after 9 months: CV% and accuracy were ≤5.9% and within 107.1–108.9% for CPT-11, ≤4.4% and within 100.3–104.9% for SN-38, ≤6.5% and within 99.0–109.1% for SN-38G and ≤14.4% and within 97.1–111.3% for APC (S3 Table).

Pharmacokinetic study

**Fig 5:**
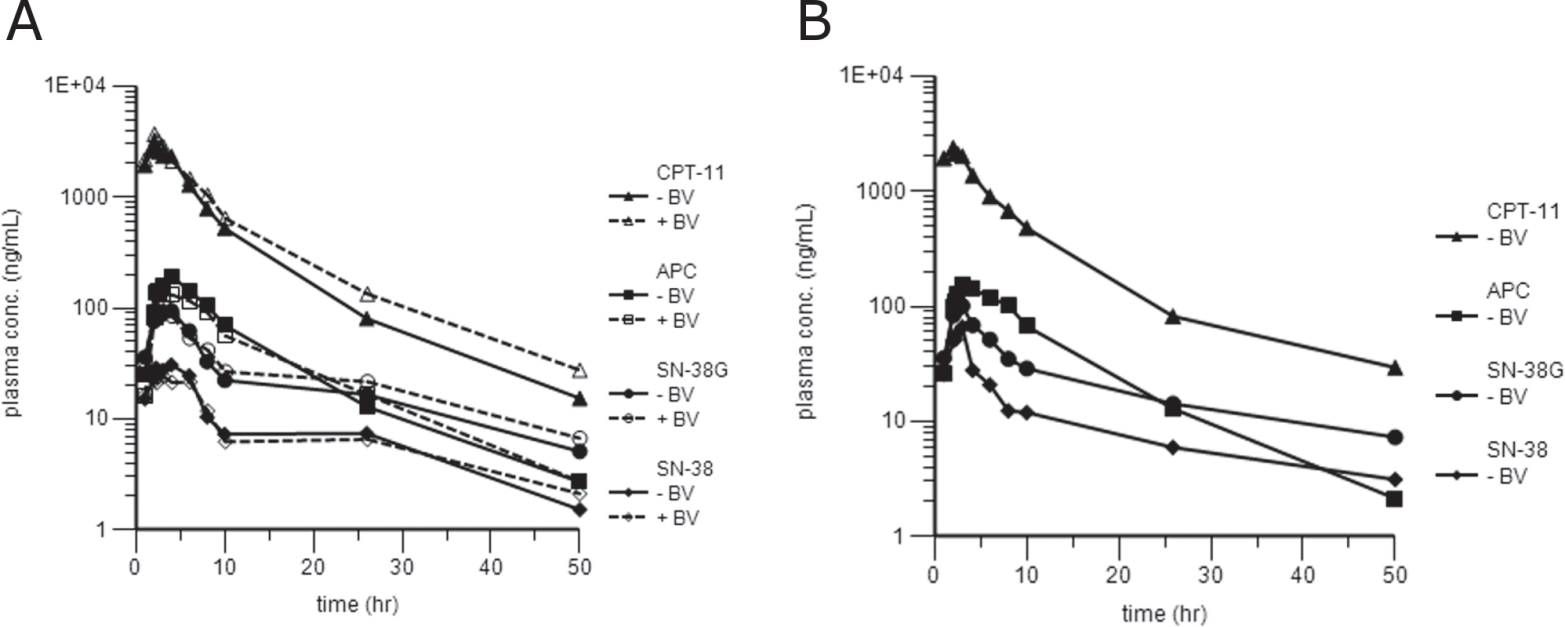
Plasma concentration-versus-time profiles of CPT-11 and its main metabolites: SN-38, SN-38G and APC. Panel A: Plasma concentration-*versus*-time profiles of CPT-11, SN-38, SN-38G and APC in one patient receiving 310 mg/m^2^ of irinotecan during the I and the II administration of the first therapy cycle; Panel B: Plasma concentration-*versus*-time profiles of CPT-11, SN-38, SN-38G and APC in one patient receiving 370 mg/m^2^ of irinotecan during the I administration of the first therapy cycle. In order to define the pharmacokinetic interactions between CPT-11 and bevacizumab (BV), the pharmacokinetic profile of CPT-11 was evaluated in absence and presence of BV in the same patient. The pharmacokinetic profile of CPT-11 alone was assessed at the first chemotherapy treatment in which BV was administered on day 3 (50 h after the start of CPT-11 infusion). Whereas, irinotecan pharmacokinetics in combination with BV was performed during the second treatment of the first cycle, when BV was administered before CPT-11 dosage.

The present method was successfully applied to a pharmacokinetic study in patients with metastatic CRC enrolled in a genotype-guided phase I study of irinotecan administered in combination with 5-FU/leucovorin (FOLFIRI) and bevacizumab. We applied this method to quantify all the samples collected by the forty-seven patients enrolled in the phase I study. For all the quantifications of each patient, the highest concentrations found were within the dynamic range of the assay without the need for further dilution steps even if the independence of analysis from the dilution was previously assessed at the dilution factors of 1:10 and 1:100 (data not shown). The data concerning the total amount of the enrolled patients will be presented in a proper pharmacokinetic study that will be published in the future. Anyway, in order to demonstrate the applicability to real samples of this method, the data related to two patients are discussed in this paper. Fig. 5 (Panel A and B) shows the plasma concentration-versus-time curves of irinotecan, SN-38, SN-38G and APC determined, during the first cycle of therapy, using the method described before, in two patients receiving respectively 310 (Panel A) and 370 (Panel B) mg/m^2^ of irinotecan as a 2-h continuous intravenous infusion. Panel A shows two pharmacokinetic profiles for each analyte because the patient received both the administrations of the first cycle whereas Panel B shows just one profile (referring to the first treatment) for analyte because the patient experienced Dose Limiting Toxicities (DLTs). Therefore, he received additional treatment with a 25% reduction in the dose of irinotecan and, consequently, blood samples were not drawn during the second treatment. The principal pharmacokinetic parameters related to the patient treated with 310 mg/m^2^ of irinotecan were as follow: Cmax and the experimental AUC (AUCexp, area under the curve of irinotecan and its main metabolites plasma concentration vs. time from 0 to 50 h) were 3003.21 ng/mL and 21298.29 ng/mL h for CPT-11, 30.70 ng/mL and 415.40 ng/mL h for SN-38, 94.36 ng/mL and 1128.51 ng/mL h for SN-38G and 194.08 ng/mL and 1994.79 ng/mL h for APC for the first administration and 3682.62 ng/mL and 25613.15 ng/mL h for CPT-11, 24.12 ng/mL and 375.22 ng/mL h for SN-38, 104.41 ng/mL and 1304.04 ng/mL h for SN-38G and 131.36 ng/mL and 1714.92 ng/mL h for APC for the second administration. CPT-11 was detectable for up to 50 h at levels 1.5 (I administration) to 3 (II administration) times the LLOQ. The terminal elimination half-life of irinotecan and its main metabolites were: 8.0 h for CPT-11, 16.8 h for SN-38, 18.3 h for SN-38G and 8.7 h for APC for the I administration and 8.9 h for CPT-11, 24.3 h for SN-38, 19.4 h for SN-38G and 9.2 h for APC for the II administration. As regards the patient treated at 370 mg/m^2^, Cmax and the AUCexp were 2370.47 ng/mL and 17759.90 ng/mL h for CPT-11 and respectively 65.95 ng/mL (reached at 3 h after the start of irinotecan infusion) and 515.52 ng/mL h for SN-38. In comparison with the patient treated at 310 mg/m^2^, the Cmax of SN-38 was more than double (65.95 vs. 30.70 ng/mL) and the AUCexp was almost 25% higher (515.42 h vs. 415.40 ng/mL). This could explain the development of DLTs as irinotecan is just a pro-drug rapidly converted to the active metabolite: SN-38. Cmax and the AUCexp were 100.72 ng/mL and 1130.06 ng/mL h for SN-38G and 155.16 ng/mL and 1817.06 ng/mL h for APC. CPT-11 was detectable for up to 50 h at levels 3 times the LLOQ. The terminal elimination half-life of irinotecan and its main metabolites were: 10.27 h for CPT-11, 21.04 h for SN-38, 19.07 h for SN-38G and 8.14 h for APC. For both the patients, irinotecan plasma concentrations appeared to decline in a bi-exponential manner, with a rapid initial phase and an extended terminal phase. Looking at the pharmacokinetic profiles, it is possible to observe that APC presents a curve very similar to irinotecan while the two regarding SN-38 and SN-38G show the same multi-exponential manner to decline with a very prolonged terminal phase.

## Conclusions

Irinotecan is widely used in clinical practice in several therapeutic regimens that employ different irinotecan dosages (125–350 mg/m^2^). The great inter-patient variability in irinotecan PK and, consequently, in response and toxicity to the treatment, still remains the major concern. To optimize irinotecan treatment, TDM, aiming at individually adjusting the dose, is required. The described bioanalytical method, based on simple protein precipitation and HPLC–MS/MS determination, quantifies irinotecan and its main metabolites in human plasma. The method, which has been successfully validated according to the FDA-EMA guidance on bioanalytical method validation, requires 100 μL of plasma, is rapid, selective, highly sensitive, precise and accurate. It has been used to measure plasma concentrations of CPT-11, SN-38, SN-38G and APC providing pharmacokinetic profiles of all these analytes, in samples from cancer patients enrolled in a phase I clinical trial. The aims of this phase I trial are the definition, on the basis of *UGT1A1* genotype, of the maximally-tolerated dose of irinotecan in FOLFIRI plus bevacizumab as first-line therapy in metastatic CRC patients and to define whether bevacizumab alters irinotecan PK. Furthermore, the identification and quantification of SN-38G add the possibility to investigate the correlation between polymorphic status of the *UGT* genes and the efficacy of glucuronidation in large patients cohorts.

## Supporting Information

S1 Table Short term stability of CPT-11 and its main metabolites in human plasma samples. (DOCX)

S2 Table Stability of CPT-11 and its main metabolites, in human plasma samples, after 2 freeze-thaw cycles and after 4 months of storage at -80°C. (DOCX)

S3 Table Stability of the working solutions of CPT-11 and its main metabolites stored at-80°C over 9 months. (DOCX)

## References

[1] Newton KF, Newman W, Hill J (2012) Review of biomarkers in colorectal cancer. Colorectal Dis 14: 3–17. 10.1111/j.1463-1318.2010.02439.x 21040359

[2] Wu AHB, Yeo KTJ (2011) Pharmacogenomic testing in current clinical practice: implementation in the clinical laboratory. New York: Humana Press 10.1080/17437199.2011.587961

[3] Toffoli G, Cecchin E, Gasparini G, D’Andrea M, Azzarello G (2010) Genotype-driven phase I study of irinotecan administered in combination with fluorouracil/leucovorin in patients with metastatic colorectal cancer. J Clin Oncol 28(5): 866–871. 10.1200/JCO.2009.23.6125 20038727 PMC4872310

[4] Liu X, Cheng D, Kuang Q, Liu G, Xu W (2013) Association between UGT1A1*28 Polymorphisms and Clinical Outcomes of Irinotecan-Based Chemotherapies in Colorectal Cancer: A Meta-Analysis in Caucasians. PLoS One 8(3): e58489 10.1371/journal.pone.0058489 23516488 PMC3597733

[5] Innocenti F, Undevia SD, Iyer L, Chen PX, Das S, et al (2004) Genetic variants in the UDP-glucuronosyltransferase 1A1 gene predict the risk of severe neutropenia of irinotecan. J Clin Oncol 22: 1382–1388. 15007088 10.1200/JCO.2004.07.173

[6] Toffoli G, Cecchin E, Corona G, Russo A, Buonadonna A, et al (2006) The role of UGT1A1*28 polymorphism in the pharmacodynamics and pharmacokinetics of irinotecan in patients with metastatic colorectal cancer. J Clin Oncol 24: 3061–3068. 16809730 10.1200/JCO.2005.05.5400

[7] Sparreboom A, de Bruijn P, de Jonge MJ, Loos WJ, Stoter G, et al (1998) Liquid chromatographic determination of irinotecan and three major metabolites in human plasma, urine and feces. J Chromatogr B Biomed Sci Appl 712(1–2): 225–35. 9698245 10.1016/s0378-4347(98)00147-9

[8] Owens TS, Dodds H, Fricke K, Hanna SK, Crews KRJ (2003) High-performance liquid chromatographic assay with fluorescence detection for the simultaneous measurement of carboxylate and lactone forms of irinotecan and three metabolites in human plasma. Chromatogr B Analyt Technol Biomed Life Sci 788(1): 65–74. 10.1016/s1570-0232(02)01016-4 12668072

[9] Sai K, Kaniwa N, Ozawa S, Sawada J (2002) An analytical method for irinotecan (CPT- 11) and its metabolites using a high-performance liquid chromatography: parallel detection with fluorescence and mass spectrometry. Biomed Chromatogr 16: 209–218. 11920947 10.1002/bmc.137

[10] Corona G, Elia C, Casetta B, Toffoli G (2010) Fast liquid chromatography–tandem mass spectrometry method for routine assessment of irinotecan metabolic phenotype. Ther Drug Monit 32(5): 638–646. 10.1097/FTD.0b013e3181ec3bf5 20683392

[11] U.S. Food and Drug Administration Center for Drug Evaluation and Research (CDER) (2001) FDA Guidance for Industry Bioanalytical Method Validation. Available: http://www.fda.gov/downloads/Drugs/GuidanceComplianceRegulatoryInformation/Guidances/UCM070107.pdf. Accessed 7 October 2014.

[12] U.S. Food and Drug Administration Center for Drug Evaluation and Research (CDER) (2013) FDA Guidance for Industry Bioanalytical Method Validation draft guidance. Available: http://www.fda.gov/downloads/Drugs/GuidanceComplianceRegulatoryInformation/Guidances/UCM368107.pdf. Accessed 7 October 2014.

[13] European Medicines Agency Committee for Medicinal Products for Human Use (CHMP) (2011) Guideline on bioanalytical method validation. Available: http://www.ema.europa.eu/docs/en_GB/document_library/Scientific_guideline/2011/08/WC500109686.pdf. Accessed 7 October 2014.

[14] Kaye B, Clark MW, Cussans NJ, Macrae PV, Stopher DA (1992) The sensitive determination of abanoquil in blood by high-performance liquid chromatography/atmospheric pressure ionization mass spectrometry. Biol Mass Spectrom 21(11): 585–9. 1360817 10.1002/bms.1200211110

[15] Fast DM, Kelley M, Viswanathan CT, O’Shaughnessy J, King PS, et al (2009) Workshop report and follow-up—AAPS Workshop on current topics in GLP bioanalysis: Assay reproducibility for incurred samples—implications of Crystal City recommendations. AAPS J 11(2): 238–241. 10.1208/s12248-009-9100-9 19381839 PMC2691460

