# Supplementary material for: Development and Validation of a High-Performance Liquid Chromatography–Tandem Mass Spectrometry Method for the Simultaneous Determination of Irinotecan and Its Main Metabolites in Human Plasma and Its Application in a Clinical Pharmacokinetic Study
Source: PLoS One. 2015 Feb 17;10(2):e0118194. doi: 10.1371/journal.pone.0118194 (PMC4331511; doi:10.1371/journal.pone.0118194)
Supplement: S1 Table — (DOCX) [file pone.0118194.s001.docx]

**Table S1.** **Short term stability of CPT-11 and its main metabolites in human plasma samples.**

|  |  | **T = 2h** | | | **T = 96h in autosampler (4°C)** | | |
| --- | --- | --- | --- | --- | --- | --- | --- |
| **Analytes** | **Nominal conc. (ng/mL)** | **Mean ± SD** | **Prec. %** | **Acc. %** | **Mean ± SD** | **Prec. %** | **Acc. %** |
| **CPT-11** | 25.00 | 23.93 ± 2.83 | 11.8 | 95.7 | 22.71 ± 0.80 | 3.5 | 90.8 |
|  | 6000.00 | 5983.16 ± 268.62 | 4.5 | 99.7 | 5443.19 ± 270.24 | 5.0 | 90.7 |
|  | 9000.00 | 8645.98 ± 303.46 | 3.5 | 96.1 | 8337.48 ± 963.60 | 11.6 | 92.6 |
| **SN38** | 2.00 | 1.96 ± 0.02 | 1.2 | 98.1 | 1.89 ± 0.25 | 13.4 | 94.6 |
|  | 150.00 | 154.21 ± 2.16 | 1.4 | 102.8 | 136.06 ± 7.51 | 5.5 | 90.7 |
|  | 400.00 | 402.43 ± 17.77 | 4.4 | 100.6 | 360.09 ± 26.21 | 7.3 | 90.0 |
| **SN-38 G** | 2.00 | 1.90 ± 0.02 | 1.1 | 95.2 | 1.80 ± 0.11 | 6.1 | 90.2 |
|  | 150.00 | 147.04 ± 2.68 | 1.8 | 98.0 | 127.55 ± 0.84 | 0.7 | 85.0 |
|  | 400.00 | 379.76 ± 24.29 | 6.4 | 94.9 | 360.82 ± 34.01 | 9.4 | 90.2 |
| **APC** | 2.00 | 2.08 ± 0.19 | 8.9 | 103.9 | 1.84 ± 0.12 | 6.8 | 91.9 |
|  | 2000.00 | 1864.57 ± 86.08 | 4.6 | 93.2 | 1715.93 ± 13.10 | 0.8 | 85.8 |
|  | 4000.00 | 3955.31 ± 235.24 | 5.9 | 98.9 | 3716.48 ± 424.53 | 11.4 | 92.9 |
