# Supplementary material for: Development and Validation of a High-Performance Liquid Chromatography–Tandem Mass Spectrometry Method for the Simultaneous Determination of Irinotecan and Its Main Metabolites in Human Plasma and Its Application in a Clinical Pharmacokinetic Study
Source: PLoS One. 2015 Feb 17;10(2):e0118194. doi: 10.1371/journal.pone.0118194 (PMC4331511; doi:10.1371/journal.pone.0118194)
Supplement: S2 Table — (DOCX) [file pone.0118194.s002.docx]

**Table S2.** **Stability of CPT-11 and its main metabolites, in human plasma samples, after 2 freeze-thaw cycles and after 4 months of storage at -80°C.**

|  |  | **After 2 freeze-thaw cycles** | | | **Stored at -20ºC over 4 months** | | |
| --- | --- | --- | --- | --- | --- | --- | --- |
| **Analytes** | **Nominal conc. (ng/mL)** | **Mean ± SD** | **Prec. %** | **Acc. %** | **Mean ± SD** | **Prec. %** | **Acc. %** |
| **CPT-11** | 25.00 | 21.67 ± 0.12 | 0.6 | 86.7 | 25.55 ± 2.53 | 9.9 | 102.2 |
|  | 6000.00 | 5853.32 ± 237.66 | 4.1 | 97.6 | 5941.81 ± 322.04 | 5.4 | 99.0 |
|  | 9000.00 | 8381.75 ± 445.81 | 5.3 | 93.1 | 8496.65 ± 605.20 | 7.1 | 94.4 |
| **SN38** | 2.00 | 2.11 ± 0.06 | 2.8 | 105.4 | 2.07 ± 0.17 | 8.3 | 103.6 |
|  | 150.00 | 151.97 ± 9.86 | 6.5 | 101.3 | 154.76 ± 3.98 | 2.6 | 103.2 |
|  | 400.00 | 412.38 ± 16.19 | 3.9 | 103.1 | 374.05 ± 30.58 | 8.2 | 93.5 |
| **SN-38 G** | 2.00 | 1.81 ± 0.05 | 2.8 | 90.5 | 1.84 ± 0.16 | 8.6 | 92.0 |
|  | 150.00 | 136.10 ± 3.72 | 2.7 | 90.7 | 130.48 ± 2.60 | 2.0 | 87.0 |
|  | 400.00 | 352.52 ± 11.30 | 3.2 | 88.1 | 340.69 ± 0.28 | 0.1 | 85.2 |
| **APC** | 2.00 | 1.91 ± 0.21 | 11.0 | 95.3 | 2.09 ± 0.17 | 8.3 | 104.5 |
|  | 2000.00 | 1891.29 ± 160.58 | 8.5 | 94.6 | 1870.85 ± 136.92 | 7.3 | 93.5 |
|  | 4000.00 | 3791.48 ± 280.50 | 7.4 | 94.8 | 3699.92 ± 254.86 | 6.9 | 92.5 |
