# Supplementary material for: Development and Validation of a High-Performance Liquid Chromatography–Tandem Mass Spectrometry Method for the Simultaneous Determination of Irinotecan and Its Main Metabolites in Human Plasma and Its Application in a Clinical Pharmacokinetic Study
Source: PLoS One. 2015 Feb 17;10(2):e0118194. doi: 10.1371/journal.pone.0118194 (PMC4331511; doi:10.1371/journal.pone.0118194)
Supplement: S3 Table — (DOCX) [file pone.0118194.s003.docx]

**Table S3.** **Stability of the working solutions of CPT-11 and its main metabolites stored at -80ºC over 9 months.**

|  |  | **Stored at -80ºC over 9 months** | | |
| --- | --- | --- | --- | --- |
| **Analytes** | **Nominal conc. (ng/mL)** | **Mean ± SD** | **Prec. %** | **Acc. %** |
| **CPT-11** | 25.00 | 26.78 ± 1.08 | 4.0 | 107.1 |
|  | 6000.00 | 6533.20 ± 388.29 | 5.9 | 108.9 |
|  | 9000.00 | 9798.46 ± 396.30 | 4.0 | 108.9 |
| **SN38** | 2.00 | 2.01 ± 0.06 | 2.8 | 100.3 |
|  | 150.00 | 154.57 ± 6.09 | 3.9 | 103.0 |
|  | 400.00 | 419.47 ± 18.31 | 4.4 | 104.9 |
| **SN-38 G** | 2.00 | 1.98 ± 0.05 | 2.5 | 99.0 |
|  | 150.00 | 149.18 ± 9.72 | 6.5 | 99.5 |
|  | 400.00 | 436.51 ± 21.69 | 5.0 | 109.1 |
| **APC** | 2.00 | 1.94 ± 0.28 | 14.4 | 97.1 |
|  | 2000.00 | 2058.05 ± 74.43 | 3.6 | 102.9 |
|  | 4000.00 | 4451.13 ± 107.37 | 2.4 | 111.3 |
